# Attitude of Syrian medical specialty trainees toward providing health care services to patients with mental disorders

**DOI:** 10.1186/s13690-023-01132-0

**Published:** 2023-08-03

**Authors:** Hidar Alibrahim, Haidara Bohsas, Sarya Swed, Yasmeen Abouainain, Zain Alabdeen Othman, Yazan Khair Eldien Jabban, Amine Rakab, Wael Hafez, Sherihan fathey, Mohammad Badr Almoshantaf, Mohamad Al Ibrahim, Bisher Sawaf, Shiekh shoib, Rama Reslan, Nour abd allatif saoud, Riham Abodest, Carlos Schönfeldt-Lecuona, Mohamed EG. Elsayed

**Affiliations:** 1 Faculty of Medicine, Aleppo University, Aleppo, Syria; 2 Faculty of Medicine, University of Jordan, Amman, Jordan; 3 Faculty of Dentistry, Albaath University, Homs, Syria; 4 Faculty of Medicine, Damascus University, Damascus, Syria; 5 Weill Cornell Medical College, Doha, Qatar; 6 NMC Royal Hospital, Abu Dhabi, UAE; 7 Department of Internal Medicine, The National Research Centre, Cairo, Egypt; 8 Department of Health, Giza, Egypt; 9 Ibn Al-Nafees Hospital, Damascus, Syria; 10 Faculty of Technical Engineering, Aleppo University, Aleppo, Syria; 11 Department of Internal Medicine, Syrian Private University, Damascus, Syria; 12 JLNM Hospital, Rainawari, Srinagar, India; 13 Directorate of Health Services, J&K, Kashmir, India; 14 Faculty of Medicine, Tishreen University, Lattakia, Syria; 15 Department of Psychiatry and Psychotherapy III, University of Ulm, Ulm, Germany; 16 Department of Psychiatry, School of Medicine and Health Sciences, Carl Von Ossietzky University Oldenburg, Oldenburg, Germany

## Abstract

**Background:**

The stigma associated with mental diseases in the healthcare system and among healthcare professionals has been identified as a significant barrier to treatment and rehabilitation and to the provision of substandard physical care for persons with mental illnesses. The goal of this study is to assess the attitude of physicians in Syria towards individuals with mental health disorders.

**Methods:**

An online cross-sectional survey was conducted among phyisicians in Syria to evaluate their attitudes toward patients with mental health disorders and their provided treatment in the time period between August 16 and October 1, 2022. The questionnaire for the study was developed based on previous research, and the inclusion criteria for the sample were all medical specialist trainees from all specialties and residents who had direct contact with people suffering from mental health disorders. The questionnaire was divided into two sections; the first included sociodemographic data on the participants and the second assessed physician’s attitudes toward mental illness patients. With the IBM SPSS V. 28.0 package tool (IBM Corporation, Armonk, NY, USA), descriptive and multivariate logistic regression analyses were used to analyze the data.

**Results:**

539 medical residents participated in this research; their average age was 26.11 (+- 1.74) years, and 50.27% were males. City residents had the highest stigma score on the third question (2.66 ± 1.06, *P* value < 0.05) in the ‘social distance’ domain. The mean stigma scores for these three items in the recovery area were (2.76 ± 1.15, 2.51 ± 0.92, and 3.73 ± 0.83), respectively, for city residents. In the ‘social distance’ domain, the stigma score of two questions (the first and fourth questions) was associated with the resident’s specialty, with dermatology residents having the highest mean score in both questions (mean = 3.6 ± 1.12, 3.43 ± 1.19, respectively). Only the second item in the ‘Detection’ domain was scored higher (mean = 3.850.81) by surgery residents than other residents. The stigma in the ‘Recovery’ domain was greatest among dermatology residents (mean = 3.710.94) than among other residents. There was a statistically significant relationship between residency and the Detection stigma scale (*p* = 0.03, Adj R2 = 0.008). There was a moderate correlation (Adj R2 = 0.048) between the Recovery scale and three of the six predictors (location, marital status, and the number of years living in the current residence). Two demographic factors (country of residence and marital status) were significantly correlated (*p*0.05) with the Social Responsibility Scale, and the Adjusted R-Squared Value was 0.006.

**Conclusion:**

Our findings indicate substantial stigma among resident physicians who treat patients with mental illnesses, which might negatively impact both the efficacy of therapy and the phyisician’s mental health. It is important to educate medical residents on mental health issues so that they can treat their patients appropriately. It is suggested that mental health concerns be included in the curriculum of residency programs for physicians so that they have adequate perspectives and attitudes about treating these patients.

**Table:** 

Text box 1. Contributions to literature
1-This study assesses the attitude of physicians in Syria towards individuals with mental health disorders and how they treat these vulnerable groups.
2-Internal medicine, pediatrics, surgery, dermatology, and ophthalmology residents have a more stigmatizing attitude.
3-Psychiatric residents have a less stigmatizing attitude toward providing health care services for patients with mental illness.
4-Anti-stigma interventions should be thought of as a pathway to change the way medical specialty trainees feel about helping other people with mental illness to recieve health care.

## Background

Stigma is a mark of shame that separates the affected individual from others and often causes them to feel excluded. It is a multifaceted process that includes many elements, such as preconceptions, identified differences, status loss and discrimination, alienation, and emotional response [1]. Mental health disorders are one of the most common issues related to stigma, including public stigma and self-stigma [2]. People with mental health disorders may face segregation, such as when someone makes a critical remark about their psychological maladjustment or therapy. Mental health patients often struggle with the symptoms and cognitive limitations caused by these illnesses and the stigmatizing attitudes of others [3]. The stigma associated with mental disorders has been identified as a major impediment to treatment and rehabilitation, as well as poor quality physical care for those suffering from mental illnesses [4]. Feeling omitted from discussions, experiencing hidden threats of coercive treatment, being forced to wait unnecessarily long while seeking care, being provided inadequate information about one’s disease or possible treatments, being treated paternally or demandingly, being told they would never get better, and being talked to or about using stigmatizing language are vital themes. Additionally, stigma affects the behaviors of health professionals who seek treatment and adversely influences their working environment. Individuals with mental diseases, such as personality disorders, are often rejected by healthcare workers and are generally seen as difficult, and manipulative [5]. One of the most common causes of stigma-related behavior among medical staff is a lack of awareness and unconscious biases, which acknowledge the power of hidden beliefs and attitudes. On the other hand, it appears that there is a connection between stigma and inadequacy of training and expertise, where it is thought that it contributes to emotions of anxiety or dread, avoidance, and clinical distance among physicians, which may significantly affect patient-provider relationships and the quality of treatment [4, 6]. Over one million Syrians are thought to have significant mental health disorders, while five million are thought to have mild to moderate mental health issues [7]. The World Health Organization reports that 3 million Syrians sought psychosocial therapy for mental health disorders in 2013, and that proportion is expected to grow each year [3]. In Syria, the stigma associated with seeking treatment at a clinic for mental health disorders impedes mental health services [7]. Even though there was a considerable gap between mental problems and access to suitable care, a combination of challenges, such as stigma, denial, and inadequate mental health literacy, contributed to the fact that the real need for treatment was not recognized [8]. The widespread occurrence of mental illnesses in Syrian society and the detrimental effects of stigma toward this particular patient group on the evaluation and treatment of these illnesses, on the other hand, motivated us to perform this cross-sectional. We performed this cross-sectional study to assess the attitude of physicians in Syria towards individuals with mental health disorders and how they treat these vulnerable groups.

## Methods

### Study design and setting

We performed this cross sectional survey in Syria between August 16 and October 1, 2022, to assess the attitude of Syrian medical specialty trainees toward providing health care services to patients with mental disorders. All medical specialist trainees from all specialties were eligible, especially those who have direct contact with patients suffering from mental illnesses. However, we excluded other medical staff members, non-Syrian medical trainees, and those who refused to participate. All respondents were aware of the objectives of the study and the name of the research team, and they could withdraw from participating at any stage. Only complete responses will be recorded and enrolled in the data analysis. Moreover, we made sure to save the data in a protected and private database. Regarding the poll tool, we relied on a previously published study to get a comprehensive, validated questionnaire [9]. To ensure proper comprehension, this questionnaire was translated into Arabic. We have collected information from respondents using convention and snowball tactics. Interesting security, a Google Form questionnaire was created and sent to respondents via social media platforms like Facebook, WhatsApp, and Telegram. Hospitals, clinics, and other health care centers were available for data collection. The smallest sample size was determined by using a single population proportion formula [*n* = [(Za/2)2. P (1-P)]/d2]. with a 95% confidence level (Z a/2 = 1.96), a 5% margin of error, *P* = the mean total stigma score for mental illness in the participants (61.36%) [9], and adding 5% for the non-response rate. The final size of the sample was 366.

### Measures

The questionnaire was split in two main components, which are as follows:

### Sociodemographic variables and work-related characteristics

In this section, we asked about age, gender, residency program, marital status, number of shifts per month, and self-report of personal experience with mental illness, family history of psychiatric disorder, violence, or serious personal problems.

### Assessment actual behaviours and attitudes toward mental illness patients

We employed a self-report survey with 20 questions called the Opening Minds Scale for Healthcare Providers (OMS-HC). This scale has five different dimensions: items, 3, 16, 17, and 19 asked about social distance, items 2 and 15 assessed other concepts like overshadowing of detection and danger; however, items 4, 5, 6, 7, and 10 concerned detections, whereas 8, 9, and 14 were recovery-related questions. Finally, Items 11, 12, 13, 18, and 20 asked about social responsibility. Reverse coding is required for items 3, 8, 9, 10, 11, 15, and 19. Each question has five possible responses, rated from 1 to 5 (strongly agree, agree, neither agree nor disagree, disagree, strongly disagree). According to this scale, the least stigmatizing score was 20, and the most stigmatizing score was 100.

### Pilot study

To demonstrate the validity and clarity of the questionnaire, we submitted it to 50 randomly selected members of the medical specialist trainee program from all specialties. In response, we adjusted the study. Our next step was to pilot a test with 50 people to determine the validity of the survey. We put out the questionnaire after doing a pilot study and making sure it was internally consistent (Cronbach's alpha level was between 0.712 and 0.861).

### Ethical consideration

The Syrian Ethical Association approved scientific research on ethical grounds (IRB: SA-2792G). The participants provided URLs to access the Internet survey on Google’s website and asked for the first page of the study when they agreed to fill out the questionnaire. Before the participants participated, the sender was sent to the next page, and each answer was stored in a safe database on the Internet.

### Statistical analysis

The statistical analysis of the data was performed using the IBM SPSS V. 28.0 package program (IBM Corporation, Armonk, NY, USA). A p-value less than 0.05 was considered for statistical significance. Descriptive statistics and frequencies were used to express categorical variables on the sociodemographic characteristics of the parents. For the statistical analysis, we categorized the levels of knowledge into "good" and "poor" based on two modified Bloom’s cutoff criteria: 70% and 80% of the total score (i.e., if a participant answered 24 and 27 of the total 34 questions correctly, respectively). A univariate analysis using the Mann–Whitney U-test (for non-normal continuous variables), t-test (for normal distribution of continuous variables), and chi-squared test (for categorical variables) was performed to determine factors influencing the knowledge level of participants. Then, a multivariate logistic regression analysis was conducted for the variables with significance (*p* < 0.05) in the univariate analysis to evaluate the odds ratios of the factors determining the knowledge level of participants.

## Results

### Socio-demographic characteristics

This study included 539 medical residents with a mean age of 26.11(± 1.74) years. Approximately half of the residents (*n* = 271, 50.27%) were males. Regarding the specialty of the residents, the Internal Medicine specialty had the highest proportion in the sample, as 191(35.4%) Internal Medicine residents were enrolled in this study. In contrast, only 9(1.66%) were psychiatry residents. Most of the residents who participated in this study were in their first three years of their residency program (*n* = 463, 85.89%). The majority of residents (*n* = 384; 71.24%) worked 0 to 10 shifts per month. Surgery was the only specialty where those who worked 10–20 shifts per month (*n* = 60) outnumbered those who worked 0–10 shifts per month (*n* = 35). More information about the demographic data of the participants is listed in Table 1.

**Table 1:** Demographic Data

		**Pediatrics**	**Psychiatric**	**Internal medicine**	**Surgery**	**Dermatology**	**Ophthalmology**	**Other**
**Age**	26.11(1.749)							
**Gender**	Female	40(14.9)	8(3)	87(32.5)	20(7.5)	35(13.1)	13(4.9)	65(24.3)
	Male	10(3.7)	1(0.4)	104(38.4)	81(29.9)	7(2.6)	13(4.8)	55(20.3)
**Place of residence**	Countryside	10(7.9)	0	44(34.6)	27(21.3)	11(8.7)	3(2.4)	32(25.2)
	City	40(9.7)	9(2.2)	147(35.7)	74(18)	31(7.5)	23(5.6)	88(21.4)
**Monthly income**	Bad	3(16.7)	0	5(27.8)	4(22.2)	1(5.6)	0	5(27.8)
	Good	14(7.9)	2(1.1)	58(32.8)	32(18.1)	12(6.8)	11(6.2)	48(27.1)
	Middle	30(9.6)	6(1.9)	114(36.7)	60(19.3)	28(9)	15(4.8)	58(18.6)
	Excellent	3(9.1)	1(3)	14(42.4)	5(15.2)	1(3)	0	9(27.3)
**Years of residential**	0–3	44(9.5)	9(1.9)	170(36.7)	79(17.1)	35(7.6)	22(4.8)	104(22.5)
	4–6	6(8)	0	21(28)	22(29.3)	7(9.3)	3(4)	16(21.3)
**monthly shifts (per day)**	0–10	26(6.8)	8(2.1)	145(37.8)	35(9.1)	40(10.4)	26(6.8)	104(27.1)
	11–20	24(16.1)	1(0.7)	46(30.9)	60(40.3)	2(1.3)	0	16(10.7)
	> 20	0	0	0	6(100)	0	0	0

### The correlation between stigma score and demographic features (gender and residency):

In the "social distance" domain, the stigma score of only one out of five questions (the 3^rd^ question) was significantly correlated (*p* < 0.05) with the place of residence, as residents who live in the city had the highest stigma score (mean = 2.66 1.06). Regarding the recovery domain, the stigma score of all three questions in this section was statistically significant (*p* < 0.05) with the place of residence, and participants who live in the city had the highest stigma score in all these 3 questions (mean = 2.76 ± 1.15, 2.5 ± 0.92, 3.73 ± 0.83, respectively). Lastly, considering the `social responsibility domain, which consists of 5 questions, the 2^nd^ question was significantly associated (*p* < 0.05) with the gender of the resident, and males had the highest stigma score (mean = 2.23 ± 1.05). Moreover, the 5^th^ question in this section was significantly associated with the place of residence, and participants who live in the city had the highest score (mean = 3.22 ± 1.15). No statistically significant association (p > 0.05) was seen between the overall stigma score and the gender and place of residence features (Table 2).

**Table 2:** Spearman Correlation between stigma score and demographic features, *Significant at 0.05 level (*p*-value < 0.05)

Scale	Gender		Residence	
	**Female mean + SD**	**Male mean + SD**	**Countryside mean + SD**	**City mean + SD**
**social distance**				
I will be more comfortable helping a person with a physical illness over a patient with a mental illness	3.31(1.140)	3.27(1.209)	3.26(1.183)	3.30(1.173)
If a co-worker tells me that he suffers from a controlled mental illness, I would like to work with him	2.40(0.822)	2.48(0.868)	2.53(0.924)	2.42(0.820)
The best treatment for mental illness is medication	2.54(0.988)	2.66(1.127)	2.40(1.026)*	2.66(1.065)*
I do not prefer for any mentally ill person to work with children even if their illness is under control	3.20(1.219)	3.46(1.198)	3.41(1.256)	3.31(1.202)
I wouldn't mind living next to a mentally ill person	2.58(0.978)	2.37(0.908)	2.33(0.984)	2.51(0.934)
**other concepts**				
If a psychiatric patient complains of physical symptoms < such as nausea, backache and headache > I will link the cause to his mental illness	3.16(0.974)	3.23(0.974)	3.30(0.970)	3.16(0.974)
Psychiatric patients rarely pose a danger to the public	3.02(1.031)	2.87(1.001)	3.01(1.080)	2.93(0.999)
**detection**				
If I am under treatment for a mental illness, I will not tell my colleagues about it	3.24(1.097)	3.23(1.163)	3.06(1.160)	3.29(1.116)
I will be more likely to seek help in a mental illness if my healthcare provider is not associated with my workplace	3.77(0.910)	3.78(0.928)	3.72(0.881)	3.79(0.930)
I will consider myself weak if I suffer from a mental illness and cannot treat it on my own	2.44(1.088)	2.45(1.114)	2.31(1.065)	2.49(1.108)
I will not hesitate to seek help if I have a mental illness	4.18(0.779)	4.07(0.887)	4.06(0.774)	4.15(0.854)
If I had a mental illness, I would tell my friends	2.82(1.019)	2.77(1.015)	2.78(1.023)	2.80(1.016)
**recovery**				
Employers should hire someone with a controlled mental illness if it is best for the job	2.53(1.054)	2.50(1.112)	2.76(1.151)*	2.44(1.050)*
I will keep seeing the doctor even if I know that he has been treated for a mental illness	2.28(0.865)	2.32(0.916)	2.50(0.925)*	2.24(0.872)*
More than half of psychopaths are not seriously trying to get better	3.51(0.958)	3.58(0.85)	3.73(0.830)*	3.49(1.005)*
social responsibility				
It is the responsibility of health care providers to instill hope in mental patients	1.74(0.834)	1.76(0.846)	1.17(0.892)	1.76(0.823)
Despite my professional beliefs I have negative reactions to mental patients	2.22(0.891)*	2.23(1.055)*	2.20(1.057)	2.23(0.951)
I can help psychopaths a little	3.89(0.827)	3.94(0.784)	3.97(0.826)	3.90(0.799)
Healthcare providers do not need to be advocates for mental patients	2.44(0.924)	2.55(1.024)	2.42(0.921)	2.51(0.993)
I struggle to feel compassion for psychopaths]	3.22(1.170)	3.06(1.145)	2.85(1.148)*	3.22(1.150)*
**overall**	25.97(1.674)	26.25(1.814)	26.22(1.618)	26.08(1.788)

*SD* Standard deviation

### The correlation between stigma score and resident specialty

The stigma score of six out of twenty questions was statistically significantly correlated with the resident specialty. In the `social distance` domain, the stigma score of two questions (the first and fourth questions) was correlated with the resident specialty, and dermatology residents had the higher mean score in both questions (mean = 3.60 ± 1.12, 3.43 ± 1.19, respectively). Additionally, the score of the first question in the "`Other concept"` domain was significantly correlated with a resident specialty, with pediatric and `other` specialties residents having the highest scores (mean = 3.26 ± 0.89, 3.26 ± 1, respectively). The score of only the 2^nd^ question in the `Detection"` domain was associated with the resident specialty, and surgery residents had the highest score (mean = 3.85 ± 0.81). Regarding the "recovery"` domain, the score of the 3^rd^ question was correlated with the resident specialty, and the dermatology residents had the highest stigma score(mean = 3.71 ± 0.94). Moreover, the 3^rd^ question in the "Social Responsibility" domain was significantly associated with resident specialty, and psychiatric residents had the highest mean score(mean = 4 ± 1.5). Finally, the overall stigma score was significantly correlated with the residents’ specialty (*p* < 0.05), as dermatology residents had the highest stigma score(mean = 60.21 ± 5.45) and psychiatry residents had the lowest score (50.33 ± 5.80) (Table3).

**Table 3:** Correlation between stigma score and resident specialty

Scale	Specialty						
	**pediatrics**	**psychiatric**	**internal medicine**	**surgery**	**dermatology**	**ophthalmology**	**other**
**social distance**							
I will be more comfortable helping a person with a physical illness over a patient with a mental illness	3.58(1.012)*	1.78(0.972)*	3.30(1.183)*	3.34(1.125)*	3.60(1.127)*	3.42(1.127)*	3.08(1.213)*
If a co-worker tells me that he suffers from a controlled mental illness, I would like to work with him	2.40(0.833)	2.33(0.707)	2.47(0.881)	2.49(0.901)	2.55(0.889)	2.27(0.874)	2.39(0.737)
The best treatment for mental illness is medication	2.60(1.190)	3(0.707)	2.54(1.070)	2.66(1.080)	2.67(1.052)	2.69(1.052)	2.56(1.002)
I do not prefer for any mentally ill person to work with children even if their illness is under control	3.36(1.174)*	2.11(1.054)*	3.35(1.246)*	3.43(1.61)*	3.43(1.192)*	3.15(1.190)*	3.32(1.223)*
I wouldn't mind living next to a mentally ill person	2.70(0.974)	2.56(0.726)	2.37(0.936)	2.34(0.952)	3.05(1.035)	2.46(0.811)	2.45(0.897)
**other concepts**							
If a psychiatric patient complains of physical symptoms < such as nausea, backache and headache > I will link the cause to his mental illness	3.26(0.899)*	2.33(1000)*	3.21(0.968)*	3.21(0.962)*	3.05(1.058)*	3.12(0.816)*	3.26(1.008)*
Psychiatric patients rarely pose a danger to the public	3.08(0.922)	2.44(1.130)	2.93(1.039)	2.90(0.985)	3.02(1.137)	2.65(0.936)	3.03(1.012)
**detection**							
If I am under treatment for a mental illness, I will not tell my colleagues about it	3.54(1.054)	2.44(1.014)	3.20(1.120)	3.13(1.197)	3.52(1.110)	3.69(1.258)	3.11(1.044)
I will be more likely to seek help in a mental illness if my healthcare provider is not associated with my workplace	3.70(0.995)*	3.44(1.424)*	3.84(0.927)*	3.85(0.817)*	3.67(0.846)*	3.81(0.749)*	3.69(0.968)*
I will consider myself weak if I suffer from a mental illness and cannot treat it on my own	2.72(1.017)	2(0.500)	2.48(1.075)	2.33(1.132)	2.71(1.175)	2.69(1.225)	2.27(1.051)
I will not hesitate to seek help if I have a mental illness	4.16(0.766)	3.89(0.928)	4.16(0.818)	4.07(0.886)	4.10(0.759)	4(0.938)	4.17(0.857)
If I had a mental illness, I would tell my friends	2.98(1.097)	2.56(1.014)	2.77(1.005)	2.83(0.991)	3.07(0.997)	3.04(1.076)	2.59(0.992)
**recovery**							
Employers should hire someone with a controlled mental illness if it is best for the job	2.74(1.242)	1.89(0.601)	2.54(1.045)	2.51(1.083)	2.43(1.063)	2.08(0.977)	2.55(1.107)
I will keep seeing the doctor even if I know that he has been treated for a mental illness	2.26(0.965)	1.78(0.667)	2.26(0.872)	2.39(1000)	2.26(0.857)	2.31(0.838)	2.35(0.827)
More than half of psychopaths are not seriously trying to get better	3.30(0.909)*	2.33(1.118)*	3.59(1.022)*	3.59(0.862)*	3.71(0.944)*	3.42(0.902)*	3.59(0.9572)*
**social responsibility**							
It is the responsibility of health care providers to instill hope in mental patients	1.64(0.776)	1.89(0.333)	1.77(0.894)	1.80(0.895)	1.62(0.731)	1.81(0.801)	1.74(0.804)
Despite my professional beliefs I have negative reactions to mental patients	2.14(0.881)	1.67(0.707)	2.23(0.934)	2.21(1.052)	2.38(1.035)	2.31(1.050)	2.24(0.996)
I can help psychopaths a little	3.86(0.857)*	4(1.5)*	3.95(0.735)*	3.92(0.857)*	3.86(0.899)*	3.85(0.732)*	3.89(0.776)*
Healthcare providers do not need to be advocates for mental patients	2.30(0.839)	2.33(1.118)	2.46(0.982)	2.43(0.920)	2.57(0.966)	3.04(1.113)	2.55(1.003)
I struggle to feel compassion for psychopaths]	3.22(1.075)	3.56(1.333)	3.14(1.195)	3.09(1.167)	2.95(1.125)	3.23(1.032)	3.14(1.16959)
**overall**	59.54(6.25)*	50.33(5.80)*	58.53(5.75)*	58.50(5.78)*	60.21(5.45)*	59.03(7.37)*	57.98(5.86)*

^*^P-value

### Spearman correlations between the five domains of the questionnaire (Social distance, other concepts, Detection, Recovery, Social responsibility)

There were a statistically significant (*p* < 0.05) weak to moderate positive correlation between the social Distance domain and the detection, recovery, and social responsibility domains (r = 0.26, 0.18, 0.17, respectively). Furthermore, a statistically significant (*p* 0.05) moderately positive correlation (r = 0.36) was observed between the other concepts domain and the recovery domain. Lastly, there was a statistically significant (*p* < 0.05) weak positive correlation between the detection domain and the "Social Responsibility" domain(r = 0.185) (Table4).

**Table 4:** Correlations between the five domains of the questionnaire (Social distance, other concepts, Detection, Recovery, Social responsibility)

	**Social distance**	**Other concepts**	**Detection**	**Recovery**	**Social responsibility**
Social distance	1	r = 0.94**, *P*-value = 0.28	r = 0.267**, *P*-value = 0	r = 0.189**, *P*-value = 0	r = 0.175**, *P*-value = 00
Other concepts	r = 0.94*, *P*-value = 0.28	1	r = -0.069, P-value = 0.107	r = 0.361**, *P*-value = 0	r = -0.062, *P*-value = 0.149
Detection	r = 0.267**, *P*-value = 0	r = -0.069, *P*-value = 0.107	1	r = -0.060, *P*-value = 0.161	r = 0.185**, *P*-value = 0
Recovery	r = 0.189**, *P*-value = 0	r = 0.361**, *P*-value = 0	r = -0.060, *P*-value = 0.161	1	r = -0.77, P-value = 0.074
Social responsibility	r = 0.175**, *P*-value = 0	r = -0.062, *P*-value = 0.149	r = 0.185**, *P*-value = 0	r = -0.077, *P*-value = 0.074	1

^*^p-value

^**^More statistically significant

### The correlation between Stigma scales for mental illness in medical specialty trainees and their demographic characteristics

The detection stigma scale was significantly correlated with the place of residence (*p* = 0.03, Adj R2 = 0.008). Out of 6 variables, 3 predictors (place of residency, marital status, and years of residential) were significantly associated (*p* = 0, 0.01, 0.01, respectively) with the recovery scale, and the adjusted R2 was 0.048. Regarding the social responsibility scale, 2 variables (place of residence and marital status) were significantly correlated (*p* < 0.05) with this scale, and the adjusted R2 was 0.006 (Table 5).

**Table 5:** Correlation between Stigma scales for mental illness in medical specialty trainees and their demographic characteristics

Dependent variable	Predictors	B	t	*p*-value	R	R^2^	Adj R^2^
Social distance	Age	0.036	0.451	0.652	0.094	0.009	-0.002
	Gender	0.185	0.802	0.423			
	place of residence	0.273	1.006	0.315			
	marital status	-0.071	-0.393	0.694			
	residential program	-0.53	-0.859	0.391			
	Years of residential	0.113	0.983	0.326			
Other concepts	Age	-0.053	-1.266	0.206	0.104	0.011	0
	Gender	-0.066	-0.536	0.592			
	place of residence	-0.227	-1.584	0.114			
	marital status	0.091	0.954	0.340			
	residential program	0.015	0.472	0.637			
	Years of residential	0.070	1.143	0.254			
Detection	Age	-0.057	-0.779	0.436	0.137	0.019	0.008
	Gender	-0.115	-0.541	0.589			
	place of residence	0.535	2.139	0.033			
	marital status	0.41	0.245	0.806			
	residential program	-0.109	-1.932	0.054			
	Years of residential	0.48	0.454	0.650			
Recovery	Age	0.019	0.316	0.752	0.243	0.059	0.048
	Gender	0.043	0.246	0.806			
	place of residence	-0.834	-4.071	0			
	marital status	0.326	2.402	0.017			
	residential program	0.038	0.812	0.417			
	Years of residential	0.206	2.357	0.019			
Social responsibility	Age	0.003	0.042	0.967	0.131	0.017	0.006
	Gender	0.015	0.075	0.940			
	place of residence	0.499	2.128	0.034			
	marital status	-0.319	-2.050	0.041			
	residential program	0.045	0.840	0.401			
	Years of residential	-0.004	-0.042	0.966			

### Proportions of stigmatizing residents from different specialties

**Fig. 1:**
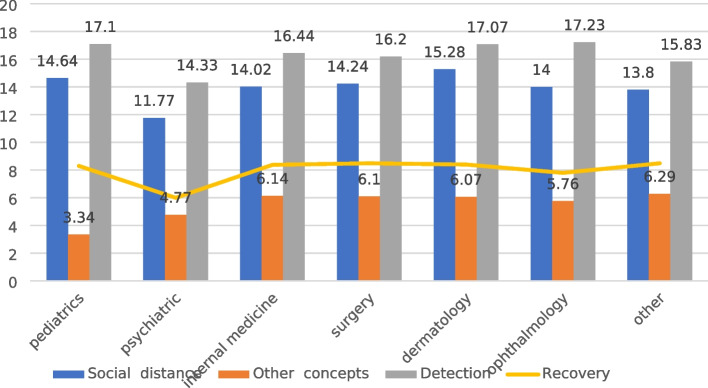
Proportions of stigmatizing residents from different specialties

15.28% of dermatology residents had a "social distance" stigma toward patients with mental illness, whereas only 11.7% of psychiatry residents had a "social distance" stigma. Regarding the "Other concepts" domain, 6.29% of residents from other specialties had a stigma toward patients with mental illness, while only 3.34% of pediatric residents had an "Other concepts" stigma. 17.23% of dermatology residents had a "detection" stigma toward mental illness patients, and only 14.33% of psychiatric residents had a "detection" stigma. Fig. 1).

## Discussion

The social stigma that surrounds mental illness has a detrimental effect on both the detection and treatment of these conditions. Access to healthcare can be limited by stigma because some people may be unwilling to seek assistance despite having mental or emotional issues because of the perception that doing demonstrates a lack of strength or competence [2, 10]. It is essential that medical experts, who are on the front line of diagnosing and treating people with mental disorders, have the right mindset when it comes to mental diseases. Therefore, we decided to conduct this cross-sectional research in order to evaluate the attitudes of physicians in Syria towards patients with mental health disorders and the delivery of treatment to vulnerable populations. This research was motivated by the high prevalence of mental health disorders in the Syrian society as well as the negative effects that stigma toward this particular patient group has on the evaluation and treatment of mental illnesses. Being a part of the Middle Eastern population may account for higher stigma ratings in our research. These higher scores might be the result of a variety of variables, such as different response styles or distinct sociocultural backgrounds. The influence of public opinion and traditional attitudes, as well as the setting in which religion is practiced, are examples of important cross-cultural distinctions that call for more research [11, 12]. According to the findings of our research, psychiatric residents are found to have a stigmatizing attitude toward patients with mental problems that is significantly lower than that of residents in other medical specialties. In comparison to the results of an Iranian study, which found that psychiatric trainees have a more accepting attitude toward patients with mental problems than their counterparts in internal medicine and cardiology, our findings found the opposite to be true. On the other hand, this discovery was of little significance for the trainees in surgery and neurology [9]. Certain medical subspecialties, including cardiology and internal medicine, have more demanding patient loads, which can lead to burnout [13]. It is possible that a more stigmatizing attitude toward mental health is connected with increased levels of both workload and burnout. It’s also possible that this is one of the reasons why psychiatric residents have fewer stigmatizing views. It is often held that individuals with mental illnesses are seen more frequently in clinics specializing in cardiology and internal medicine than in surgical specialties [14–16]. When looking at the various stigma subscales side by side, our research only demonstrates that psychiatric trainees are stigmatized less when it comes to societal duty compared to other groups. Previous research has demonstrated that a stigma attached to social duty has a detrimental impact on empathy [17]. An integrated relationship model has been proposed: physicians who have more experience, more excellent patient-to-physician contact, and more empathy toward patients with mental disorders feel less uneasy around those patients, and as a result, they tend to reduce the social distance that separates them from those patients [18]. There were a total of six different factors, however, only three of them (years of residential experience, marital status, and site of residence) had a meaningful association with the Recovery scale. In addition, while looking at the social responsibility scale, it was found that there was a substantial correlation between this scale and two other variables: location of residence and marital status. In contrast to the research carried out in Iran, it would appear that factors like age, gender, marital status, and the number of shifts had no significant bearing on the stigma associated with mental problems [9, 19]. When compared to residents in other medical specialties, those trained in psychiatry were more likely to agree that medication is the most effective form of therapy for mental illness. It was widely perceived to be more successful and valuable than pharmaceutical therapy, and in contrast to the study that was conducted in Nepal, many of the participants viewed psychoeducation and psychosocial counseling as practices that involved the offering of advice or suggestions. The use of less stigmatizing language, with counselors using terms such as "heart-mind problems" or focusing on the symptoms reported by patients rather than using the word "mental health" in the counseling sessions, is one of the possible reasons why people prefer psychological treatment over other types of treatment. Other possible reasons include the involvement of family members in the counseling sessions, home visits by the community counselors, which were less stigmatizing and more confidential, and the involvement of family members in the counseling sessions. After the treatment, significant improvements were reported in patients’ health conditions and outcomes. Subsequently, many participants were found to be engaged in their regular day-to-day activities, such as taking care of the domestic animals, involvement in farm-related activities, and other activities that generated income [20]. This is because there is a shortage of psychotropic medications in the healthcare facilities, which may be the consequence of a lengthy administrative procedure for the acquisition and distribution of medicines, which leads to frequent stock-outs of medicines. In addition, in 2015, there was a shortage of gasoline as a result of a blockage that occurred on the border between India and Nepal. This caused issues with the distribution of medications to the appropriate medical institutions for a period of several months. Problems with the regular supply of psychotropic drugs have also been observed in a variety of studies, and this is regarded as one of the most significant hurdles to the integration of mental health services into the primary health care system [20].

### Strengths and limitations

The findings of this study need to be interpreted with the following limitations taken into consideration: Because this was an online, cross-sectional survey, it is not possible to generalize these findings to all mental health practitioners in Syria, nor is it possible to establish any causal links between the variables. In addition, further difficulties arise if consumers are unable to access the internet or if a device is unable to finish an online survey. Due to the fact that the study only included residents, information on the stigma associated with other staff members, such as healthcare attendants, patient services associates, and administrative employees, was not collected and therefore may be different. However, the interpretation of our findings is hampered by the low number of people in our sample, particularly for the analyses that compare the two groups. Another drawback of our research is that it was not a longitudinal study, which would have allowed for the observation of participants over a longer period of time and the implementation of stigma-reducing treatments. Not including the residents’ own experiences with mental illness is another important exclusion since such experiences might lead to prejudice. One of the strengths is that participants have been recruited from a wide range of different fields.

## Conclusion

Internal medicine, pediatrics, surgery, dermatology, and ophthalmology residents have a more stigmatizing attitude, whereas psychiatric residents have a less stigmatizing attitude toward providing health care services for patients with mental illness. The attitude of medical specialty trainees toward providing health care services for patients with mental illness is not uniform. It would appear that not every encounter may be good for generating a better attitude about mental illness; however, this can only happen if certain preconditions are met, such as having an organized contact that results in favorable consequences. Anti-stigma interventions should be thought of as a way to change the way medical specialty trainees feel about helping people with mental illness get health care.

## Data Availability

The data are available upon request from the corresponding author.
